# Gender-specific effects of oxidative balance score on the prevalence of diabetes in the US population from NHANES

**DOI:** 10.3389/fendo.2023.1148417

**Published:** 2023-05-04

**Authors:** Cuiling Wu, Chenxia Ren, Yingda Song, Huifang Gao, Xin Pang, Lianyun Zhang

**Affiliations:** ^1^Department of Biochemistry, Changzhi Medical College, Changzhi, China; ^2^Central Laboratory, Changzhi Medical College, Changzhi, China; ^3^Department of Thoracic Surgery, Shanxi Provincial People’s Hospital, Taiyuan, China; ^4^Fifth Clinical Medical College, Shanxi Medical University, Taiyuan, China

**Keywords:** oxidative balance score, gender, diabetes, cross-sectional study, adults

## Abstract

**Background:**

The relationship between oxidative balance score (OBS) and diabetes remains poorly understood and may be gender-specific. We conducted a cross-sectional study to investigate the complex association between OBS and diabetes among US adults.

**Methods:**

Overall, 5,233 participants were included in this cross-sectional study. The exposure variable was OBS, composed of scores for 20 dietary and lifestyle factors. Multivariable logistic regression, subgroup analysis, and restricted cubic spline (RCS) regression were applied to examine the relationship between OBS and diabetes.

**Results:**

Compared to the lowest OBS quartile group (Q1), the multivariable-adjusted odds ratio (OR) (95% confidence interval (CI) for the highest OBS quartile group (Q4) was 0.602 (0.372–0.974) (*p* for trend = 0.007), and for the highest lifestyle, the OBS quartile group was 0.386 (0.223–0.667) (*p* for trend < 0.001). Moreover, gender effects were found between OBS and diabetes (*p* for interaction = 0.044). RCS showed an inverted-U relationship between OBS and diabetes in women (*p* for non-linear = 6e−04) and a linear relationship between OBS and diabetes in men.

**Conclusions:**

In summary, high OBS was negatively associated with diabetes risk in a gender-dependent manner.

## 1 Introduction

The spread of Western lifestyles has gradually increased the use of high-calorie and high-fat diets, sedentary lifestyles, and the number of adults with diabetes (1). According to the International Diabetes Federation 2019 report, the overall prevalence of diabetes among adults aged 20 to 79 years was 9.3%. This is expected to rise to 693 million worldwide by 2045 (2–4). Diabetes and its complications are life-threatening problems.

A large body of evidence demonstrated that oxidative stress plays a crucial role in the development and progression of diabetes (5). The oxidative balance score (OBS) is a comprehensive indicator containing 20 different dietary and lifestyle components, which highlights the overall balance of pro- and antioxidants at dietary and lifestyle levels. In general, a higher OBS indicates that antioxidants prevail over pro-oxidants. Numerous studies have reported a negative correlation between OBS and the incidence of different diseases, such as breast cancer (6), new-onset hypertension (7), osteoporosis (8), and leukocyte telomere length (9). However, the potential relationship between OBS and diabetes risk remains to be known.

Herein, we investigated the relationship between OBS and the prevalence of diabetes. We examined the possible effects of OBS on diabetes using data from the National Health and Nutrition Examination Survey (NHANES) from 2007 to March 2020.

## 2 Method

### 2.1 Source of data and study population

**Figure 1:**
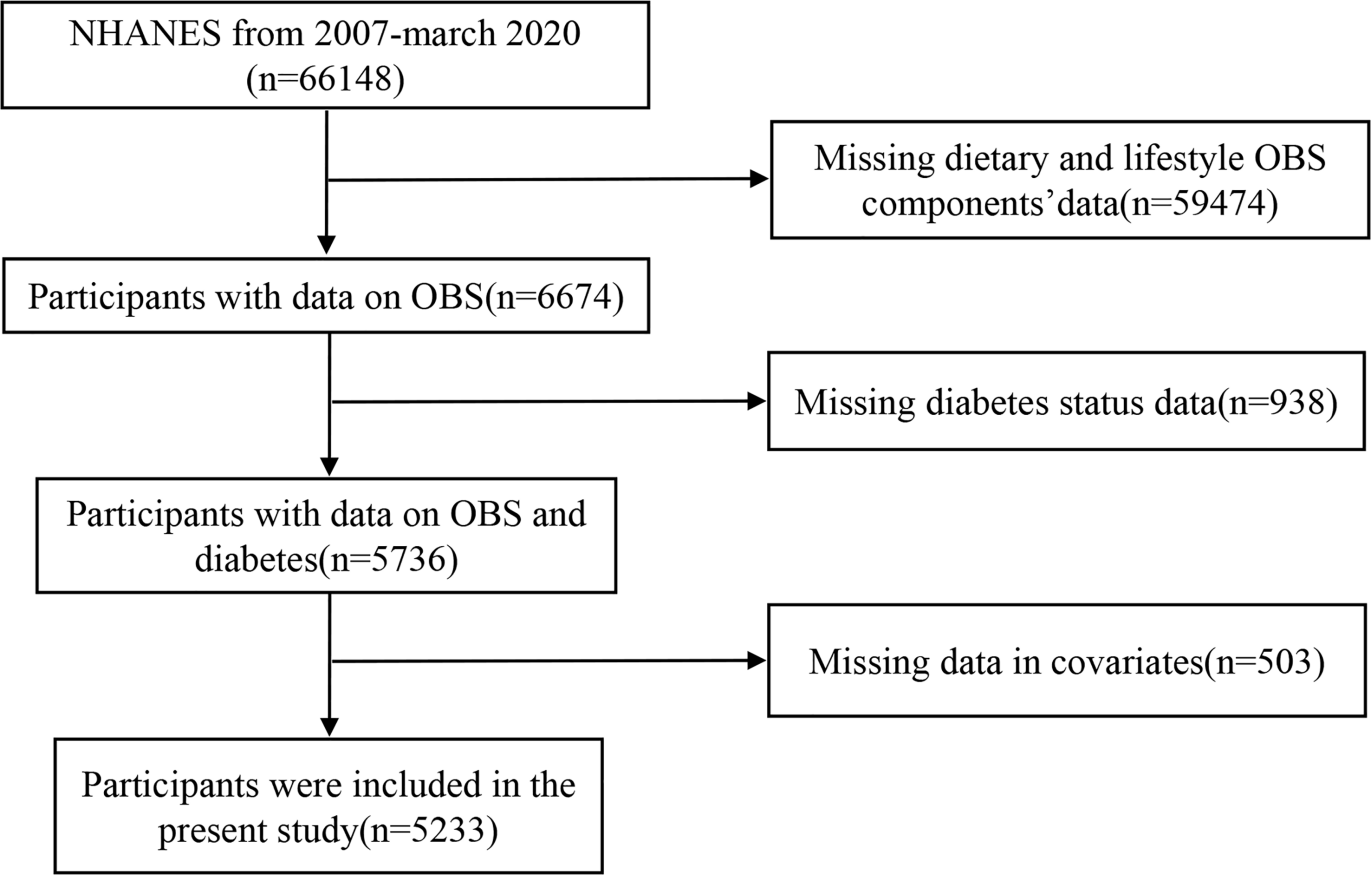
Flow diagram.

The NHANES was a national cross-sectional study assessing the health and nutrition status of adults and children in the US population. The study used a “stratified multistage probability sampling,” in which the information was collected from relevant interviews, examinations, dietary questionnaires, and laboratory measurements. In total, 5,233 participants were chosen from 2007 to March 2020. Exclusion criteria were as follows: age of participants was <20 or ≥80 years, participants without dietary or lifestyle data, participants without known diabetes status, and variables with missing values (**Figure 1**). All participants provided signed written informed consent, and the study conformed to ethical standards.

### 2.2 Calculation of the oxidative balance score

The development and calculation of the OBS have been reported previously (9). The total OBS components were assigned a score by intake, property, and gender. Overall scores were the sum of dietary and lifestyle scores. Higher OBS was positively correlated with participants’ antioxidant activity. Dietary components and lifestyle components were used to calculate OBS. Dietary components of OBS consisted of dietary fiber, carotene, riboflavin, niacin, calcium, magnesium, zinc, total folate, vitamins (B_6_, B_12_, C, and E), copper, selenium, total fat, and iron. The lifestyle components of OBS consisted of physical activity, alcohol drinking, body mass index, and cotinine. Fat, iron, alcohol drinking, cotinine, and body mass index were classified as pro-oxidants, and the remaining components were classified as antioxidants.

### 2.3 Evaluation of diabetes

The diagnostic criteria for diabetes were as follows: previous diagnosis of diabetes by a physician, glycated hemoglobin (HbA1c) >6.5%, fasting glucose ≥7.0 mmol/L, random blood glucose ≥11.1 mmol/L, 2-h oral glucose tolerance test (OGTT) ≥11.1 mmol/L, and use of diabetes medication or insulin.

### 2.4 Covariates

Based on the existing literature and clinical consideration, we selected covariates that could play roles as potential confounders in the associations between OBS and diabetes. The standardized household interviews were used to obtain the demographic characteristics, including age, gender, race, educational level, and poverty income ratio (PIR). Age was divided into three groups (20–39, 40–59, and 60–79 years), with 20–39 years as the reference. Race was divided into non-Hispanic white, non-Hispanic black, Mexican American, and others, with non-Hispanic black as the reference. Education level was graded into primary school or less, middle and high school, and college or higher, with college or higher as the reference. Poverty was defined as PIR ≤ 1.0 and divided into two categories of PIR (≤1.0 and >1.0), with PIR ≤ 1.0 as the reference. White blood cell (WBC) count, platelet (Plt) count, neutrophil (Neu) count, lymphocyte (Lym) count, and hemoglobin (Hb) level were obtained from the laboratory data. Chronic kidney disease (CKD), cardiovascular disease (CVD), hypertension, dyslipidemia, and smoking are important risk factors for diabetes. Therefore, these diseases were included in the analysis. According to the KDIGO 2021 Clinical Practice Guideline for the Management of Glomerular Diseases, albumin-to-creatinine ratio (ACR) and estimated glomerular filtration rate (eGFR) were used to define CKD. ACR ≥ 30 mg/g (3 mg/mmol) and eGFR < 60 ml/min/1.73 m^2^ were defined as diagnostic criteria of CKD. CVD was defined as congestive heart failure, coronary heart disease, heart attack, angina, and stroke. The diagnostic criteria for hypertension were as follows: average systolic blood pressure ≥140 mmHg or average diastolic blood pressure ≥90 mmHg after at least three times of measurement, use of anti-hypertensive drugs, and subject- or physician-reported diagnosis of hypertension. The diagnostic criteria for dyslipidemia were as follows: total cholesterol level ≥5.18 mmol/L, triglyceride level ≥150 mg/dl, high-density lipoprotein-cholesterol <1.04 mmol/L in men and <1.30 mmol/L in women, low-density lipoprotein-cholesterol ≥3.37 mmol/L, or the use of cholesterol-lowering drugs. Smoking status was defined into three categories: never (smoked less than 100 cigarettes in life), former (smoked more than 100 cigarettes in life and does not smoke now), and smoked more than 100 cigarettes in life and smokes some days or every day. No or never was taken as the reference for all of the above conditions.

### 2.5 Statistical analysis

Considering the complexity of the sampling method, the study subject was a weighted statistical analysis. Continuous variables are presented as mean (standard deviation (SD)), and categorical variables are summarized as frequency (percentage). For baseline characteristics, categorical variables were compared using the chi-square test, and continuous variables were compared using the t-test or one-way analysis of variance. Multivariable logistic regression models (crude models to model 3) were used to investigate the relationship between OBS and diabetes after adjusting for different potential confounders. The crude model was not adjusted for any covariates. Model 1 was adjusted for age, gender, race, and education. Model 2 was further adjusted for WBC count, Neu count, Hb count, and Plt count. Model 3 was adjusted for the variables in model 2 and additional confounders, including CKD, CVD, smoking status, hypertension, and dyslipidemia. We further assessed the heterogeneity between OBS and diabetes through subgroup analysis for the following variables: age groups, gender, race, education, CKD, CVD, hypertension, dyslipidemia, and smoking status. We applied restricted cubic spline (RCS) analysis with four knots to evaluate the non-linear associations between OBS and diabetes risk. R statistical software (version 4.2.2) was applied for all statistical analyses and mapping. Alpha was set at <0.05 for statistical significance, and all analyses were two-sided. A two-sided *p*-value <0.05 was defined as the significance threshold.

## 3 Results

### 3.1 Baseline characteristics

In total, 5,233 participants from NHANES (2007 to March 2020) were enrolled in the present study, of whom 622 (11.89%) had diabetes. Among all participants with diabetes, men (461, 11.64%) showed a higher prevalence than women (161, 5.61%). Participants with diabetes were older and had lower education levels, but PIR did not differ between the two groups (**Table 1**). The comparison of baseline characteristics showed that patients with diabetes had a higher prevalence of CKD, CVD, hypertension, and dyslipidemia. Participants without diabetes showed significantly higher OBS and lifestyle OBS when compared with participants with diabetes. There was no significant difference in Lym count (*p* = 0.7) and dietary OBS (*p* = 0.06).

**Table 1:** Baseline characteristics of all participants by diabetes.

Variables	Overall (n = 5,233)	Non-DM (n = 4,611)	DM (n = 622)	*p*-Value
Age (years)	45.23 (0.41)	44.23 (0.42)	55.11 (0.78)	<0.0001
Gender, n (%)				<0.0001
Female	2,062 (39.4)	1,901 (94.39)	161 (5.61)	
Male	3,171 (60.6)	2,710 (88.36)	461 (11.64)	
Age group, n (%)				<0.0001
20–39	2,103 (40.19)	2,023 (97.12)	80 (2.88)	
40–59	1,951 (37.28)	1,675 (89.39)	276 (10.61)	
60–79	1,179 (22.53)	913 (81.29)	266 (18.71)	
Education, n (%)				<0.001
College and higher	3,495 (66.79)	3,136 (91.79)	359 (8.21)	
Middle and high school	1,535 (29.33)	1,314 (88.92)	221 (11.08)	
Primary school and less	203 (3.88)	161 (79.08)	42 (20.92)	
Race, n (%)				0.01
Black	1,029 (19.66)	868 (87.89)	161 (12.11)	
Mexican	623 (11.91)	520 (86.97)	103 (13.03)	
Other	989 (18.9)	873 (90.43)	116 (9.57)	
White	2,592 (49.53)	2,350 (91.57)	242 (8.43)	
PIR				0.65
≤1	812 (15.52)	719 (90.23)	93 (9.77)	
>1	4,421 (84.48)	3,892 (90.94)	529 (9.06)	
WBC (×10^9^/L)	7.01 (0.05)	6.98 (0.05)	7.36 (0.14)	0.01
Neu (×10^9^/L)	4.12 (0.04)	4.08 (0.04)	4.45 (0.10)	<0.001
Lym (×10^9^/L)	2.10 (0.01)	2.11 (0.01)	2.08 (0.07)	0.7
Hb (g/L)	14.52 (0.03)	14.50 (0.03)	14.69 (0.08)	0.03
Plt (×10^6^/L)	237.68 (1.57)	238.57 (1.61)	228.78 (4.45)	0.03
CKD, n (%)				<0.0001
No	4,693 (89.68)	4,246 (92.36)	447 (7.64)	
Yes	540 (10.32)	365 (75.37)	175 (24.63)	
CVD, n (%)				<0.0001
No	4,910 (93.83)	4,390 (91.86)	520 (8.14)	
Yes	323 (6.17)	221 (73.09)	102 (26.91)	
Hypertension, n (%)				<0.0001
No	3,485 (66.6)	3,273 (95.35)	212 (4.65)	
Yes	1,748 (33.4)	1,338 (80.82)	410 (19.18)	
Dyslipidemia, n (%)				<0.0001
No	1841,(35.18)	1,747 (96.01)	94 (3.99)	
Yes	3,392 (64.82)	2,864 (88.28)	528 (11.72)	
Smoking status, n (%)				<0.001
Never	2,570 (49.11)	2,327 (92.49)	243 (7.51)	
Former	1,364 (26.07)	1,139 (87.38)	225 (12.62)	
Now	1,299 (24.82)	1,145 (91.65)	154 (8.35)	
OBS	29.69 (0.08)	29.76 (0.08)	28.99 (0.20)	<0.001
Dietary OBS	25.55 (0.06)	25.58 (0.07)	25.28 (0.16)	0.06
Lifestyle OBS	4.13 (0.04)	4.17 (0.04)	3.71 (0.09)	<0.0001

All values represented are weighted means (standard deviation) or counts (weighted percentage).

SD, standard deviation; PIR, poverty income ratio; WBC, white blood cells; Neu, neutrophil; Lym, lymphocyte; Hb, hemoglobin; Plt, platelet; CKD, chronic kidney disease; CVD, cardiovascular disease; OBS, oxidative balance score; DM, diabetes.

All 5,233 individuals were categorized into four groups according to OBS quartiles: Q1 (OBS, 5 to 28; median, 26), Q2 (OBS, 29 to 30; median, 30), Q3 (OBS, 31 to 32; median, 31), and Q4 (OBS, 32 to 36; median, 33). As the reference group, participants in the first quartile group (Q1) with lower OBS were more likely to be white and have higher educational levels and PIR. In addition, participants in the Q1 had a lower incidence of CKD, diabetes, hypertension, dyslipidemia, and smoking. Gender was not significantly different between OBS quartiles, suggesting an evenly balanced distribution of men and women in all quartiles (**Table 2**). We also performed the same analysis on dietary OBS and lifestyle OBS. Dietary OBS did not significantly affect the prevalence of diabetes, CKD, CVD, hypertension, and dyslipidemia. Increased lifestyle OBS was associated with the prevalence of diabetes, CKD, CVD, hypertension, and dyslipidemia. Similar to OBS, lifestyle OBS was associated with the prevalence of diabetes (**Supplementary Tables 1, 2**).

**Table 2:** Baseline characteristics of all participants by the OBS quartile.

Variables	Overall (n = 5,233)	Q1 (n = 1,608)	Q2 (n = 1,310)	Q3 (n = 1,422)	Q4 (n = 893)	*p*-value
Age (years)	45.23 (0.41)	43.32 (0.60)	44.62 (0.64)	46.13 (0.60)	47.31 (0.78)	<0.001
Gender, n (%)						0.1
Female	2,062 (39.4)	604 (25.23)	494 (23.55)	577 (30.41)	387 (20.81)	
Male	3,171 (60.6)	1,004 (27.72)	816 (26.18)	845 (28.22)	506 (17.88)	
Age group, n (%)						<0.001
20–39	2,103 (40.19)	681 (29.60)	537 (25.45)	545 (27.61)	340 (17.34)	
40–59	1,951 (37.28)	597 (26.93)	515 (25.96)	538 (29.31)	301 (17.80)	
60–79	1,179 (22.53)	330 (20.14)	258 (22.41)	339 (31.87)	252 (25.57)	
Education, n (%)						<0.0001
College and higher	3,495 (66.79)	854 (21.20)	827 (23.76)	1,054 (31.39)	760 (23.64)	
Middle and high school	1,535 (29.33)	677 (42.07)	425 (28.62)	319 (22.96)	114 (6.35)	
Primary school and less	203 (3.88)	77 (40.38)	58 (31.00)	49 (20.89)	19 (7.73)	
Race, n (%)						<0.0001
Black	1,029 (19.66)	499 (49.20)	269 (26.55)	192 (17.63)	69 (6.61)	
Mexican	623 (11.91)	177 (28.60)	189 (29.51)	182 (30.09)	75 (11.81)	
Other	989 (18.9)	247 (25.12)	214 (23.42)	285 (28.86)	243 (22.60)	
White	2,592 (49.53)	685 (24.32)	638 (24.81)	763 (30.34)	506 (20.53)	
PIR						<0.0001
≤1	812 (15.52)	377 (44.18)	197 (23.27)	167 (21.67)	71 (10.89)	
>1	4,421 (84.48)	1,231 (24.87)	1,113 (25.27)	1,255 (29.90)	822 (19.95)	
WBC (×10^9^/L)	7.01 (0.05)	7.51 (0.10)	7.21 (0.07)	6.81 (0.07)	6.37 (0.09)	<0.0001
Neu (×10^9^/L)	4.12 (0.04)	4.46 (0.07)	4.24 (0.05)	3.97 (0.05)	3.71 (0.07)	<0.0001
Lym (×10^9^/L)	2.10 (0.01)	2.21 (0.03)	2.16 (0.03)	2.07 (0.02)	1.93 (0.03)	<0.0001
Hb (g/L)	14.52 (0.03)	14.63 (0.06)	14.58 (0.06)	14.47 (0.05)	14.35 (0.06)	0.002
Plt (×10^6^/L)	237.68 (1.57)	243.98 (2.24)	237.34 (2.58)	237.81 (2.56)	229.12 (3.06)	0.002
DM, n (%)						0.004
No	4,611 (88.11)	1,360 (25.79)	1,156 (24.98)	1,269 (29.61)	826 (19.62)	
Yes	622 (11.89)	248 (35.61)	154 (26.12)	153 (24.37)	67 (13.89)	
CKD, n (%)						0.003
No	4,693 (89.68)	1,399 (25.90)	1,190 (24.99)	1,297 (29.85)	807 (19.26)	
Yes	540 (10.32)	209 (34.87)	120 (26.07)	125 (21.63)	86 (17.43)	
CVD, n (%)						0.65
No	4,910 (93.83)	1,485 (26.66)	1,227 (24.94)	1,348 (29.08)	850 (19.32)	
Yes	323 (6.17)	123 (27.07)	83 (27.62)	74 (30.06)	43 (15.25)	
Hypertension, n (%)						<0.0001
No	3,485 (66.6)	960 (24.48)	861 (24.30)	962 (29.26)	702 (21.95)	
Yes	1,748 (33.4)	648 (31.62)	449 (26.84)	460 (28.85)	191 (12.70)	
Dyslipidemia, n (%)						<0.001
No	1,841 (35.18)	527 (23.98)	440 (23.82)	499 (28.70)	375 (23.50)	
Yes	3,392 (64.82)	1,081 (28.05)	870 (25.72)	923 (29.35)	518 (16.88)	
Smoking status, n (%)						<0.0001
Never	2,570 (49.11)	573 (18.40)	590 (21.97)	802 (34.28)	605 (25.35)	
Former	1,364 (26.07)	345 (22.86)	345 (26.36)	407 (29.35)	267 (21.43)	
Now	1,299 (24.82)	690 (50.65)	375 (30.62)	213 (17.01)	21 (1.72)	
OBS	29.69 (0.08)	25.10 (0.13)	29.55 (0.02)	31.50 (0.02)	33.50 (0.03)	<0.0001
Dietary OBS	25.55 (0.06)	22.13 (0.15)	25.89 (0.05)	26.97 (0.04)	27.73 (0.03)	<0.0001
Lifestyle OBS	4.13 (0.04)	2.96 (0.05)	3.66 (0.05)	4.53 (0.04)	5.77 (0.03)	<0.0001

All values represented are weighted means (standard deviation) or counts (weighted percentage). The OBS was divided into four levels by quartile (5 < Q1 ≤ 28, 29 < Q2 ≤ 30, 31 < Q3 ≤ 32, and 32 < Q4 ≤36).

WBC, white blood cells; Neu, neutrophil; Lym, lymphocyte; Hb, hemoglobin; Plt, platelet; CKD, chronic kidney disease; CVD, cardiovascular disease; OBS, oxidative balance score; DM, diabetes.

### 3.2 Relationship between OBS and diabetes

The results of the multivariable logistic regressions showed that OBS was significantly associated with diabetes (**Table 3**). In model 3, the risk of diabetes decreased by 3.8% with a 1-unit increase in OBS (OR = 0.962, 95% confidence interval (CI) 0.935–0.990), revealing that OBS was negatively correlated with the risk of diabetes. Compared with participants in Q1, those in Q2, Q3, and Q4 were at lower risk of diabetes in all models. Compared with participants in Q1, subjects in Q4 had 39.8% (OR = 0.602; 95% CI, 0.378–0.974) decreased risk of diabetes in model 3. We further explored the effects of dietary OBS and lifestyle OBS on diabetes using logistic regression models. Although dietary OBS and lifestyle OBS were supposed to be protective factors for diabetes, results for dietary OBS were not statistically significant (**Supplementary Tables 3, 4**).

**Table 3:** Association of the OBS with diabetes, NHANES 2007–March 2020.

Diabetes	OR (95% CI); *p*-value							
	Crude model		Model 1		Model 2		Model 3	
Continuous	0.95 (0.93, 0.97)	<0.0001	0.94 (0.92, 0.97)	<0.0001	0.95 (0.92, 0.98)	<0.001	0.96 (0.94, 0.99)	<0.009
Q1	1.00 (ref)		1.00 (ref)		1.00 (ref)		1.00 (ref)	
Q2	0.76 (0.56, 1.02)	0.07	0.71 (0.52, 0.98)	0.04	0.73 (0.53, 1.00)	0.05	0.76 (0.55, 1.06)	0.10
Q3	0.60 (0.42, 0.85)	0.005	0.55 (0.38, 0.78)	0.001	0.58 (0.40, 0.83)	0.003	0.62 (0.43, 0.90)	0.01
Q4	0.51 (0.33, 0.79)	0.003	0.44 (0.28, 0.69)	<0.001	0.48 (0.30, 0.76)	0.002	0.60 (0.37, 0.97)	0.04
*p* for trend		<0.001		<0.0001		<0.001		0.007

The OBS was converted from a continuous variable to a categorical variable (quartiles). Data are presented as OR (95% CI). Crude model was adjusted with no covariates.

OBS, oxidative balance score; NHANES, National Health and Nutrition Examination Survey.

### 3.3 Subgroup analysis

Subgroup analysis was performed based on gender, age group, race, education, CVD, CKD, dyslipidemia, hypertension, and smoking status (**Table 4**). Statistical significance was found in gender subgroups of diabetes (*p* for interaction = 0.044). Subgroup analysis showed that the female subgroup was more sensitive to OBS compared with the male subgroup. Women had an 88.0% lower risk of developing diabetes in the fourth quartile of age compared with the first quartile. In contrast, the risk of diabetes only decreased by 38.6% in men (all *p* for trend <0.05).

**Table 4:** Subgroup analyses of the association between OBS and diabetes, NHANES 2007–March 2020.

Variables	Q1	Q2	Q3	Q4	*p* for trend	*p* for interaction
Age group						0.211
60–79	Ref	1.063 (0.637, 1.776)	1.031 (0.579, 1.833)	0.821 (0.480, 1.403)	0.456	
20–39	Ref	0.574 (0.290, 1.138)	0.334 (0.153, 0.726)	0.212 (0.067, 0.672)	0.001	
40–59	Ref	0.639 (0.416, 0.982)	0.423 (0.261, 0.684)	0.345 (0.142, 0.838)	0.001	
Gender						0.044
Male	Ref	0.838 (0.570, 1.232)	0.592 (0.410, 0.855)	0.614 (0.363, 1.041)	0.016	
Female	Ref	0.456 (0.214, 0.970)	0.468 (0.246, 0.891)	0.120 (0.036, 0.401)	<0.0001	
Race						0.832
White	Ref	0.723 (0.460, 1.136)	0.552 (0.326, 0.933)	0.520 (0.290, 0.933)	0.009	
Mexican	Ref	1.008 (0.478, 2.123)	0.665 (0.298, 1.485)	0.617 (0.152, 2.499)	0.26	
Black	Ref	0.835 (0.483, 1.446)	0.719 (0.381, 1.357)	0.186 (0.063, 0.550)	0.017	
Other	Ref	0.545 (0.236, 1.256)	0.603 (0.242, 1.502)	0.305 (0.129, 0.721)	0.029	
Education						0.187
College and higher	Ref	0.835 (0.551, 1.264)	0.696 (0.432, 1.118)	0.519 (0.304, 0.887)	0.009	
Middle and high school	Ref	0.667 (0.387, 1.149)	0.419 (0.207, 0.848)	0.626 (0.220, 1.782)	0.05	
Primary school and less	Ref	0.192 (0.044, 0.841)	0.091 (0.024, 0.345)	0.074 (0.011, 0.501)	<0.001	
CKD						0.069
No	Ref	0.598 (0.420, 0.852)	0.516 (0.344, 0.774)	0.470 (0.289, 0.764)	<0.001	
Yes	Ref	1.309 (0.599, 2.859)	0.776 (0.371, 1.624)	0.312 (0.121, 0.804)	0.011	
CVD						0.268
No	Ref	0.727 (0.526, 1.005)	0.508 (0.339, 0.760)	0.393 (0.242, 0.638)	<0.0001	
Yes	Ref	0.543 (0.201, 1.462)	0.693 (0.320, 1.500)	0.958 (0.253, 3.627)	0.872	
Smoking status						0.472
Never	Ref	0.534 (0.283, 1.007)	0.415 (0.236, 0.729)	0.353 (0.182, 0.685)	0.001	
Former	Ref	0.947 (0.532, 1.686)	0.736 (0.439, 1.236)	0.493 (0.254, 0.955)	0.015	
Now	Ref	0.621 (0.334, 1.154)	0.285 (0.147, 0.551)	0.203 (0.070, 0.590)	<0.001	
Hypertension						0.121
No	Ref	0.436 (0.256, 0.744)	0.538 (0.298, 0.971)	0.470 (0.229, 0.962)	0.038	
Yes	Ref	1.022 (0.694, 1.506)	0.611 (0.417, 0.894)	0.601 (0.334, 1.082)	0.01	
Dyslipidemia						0.239
Yes	Ref	0.675 (0.480, 0.949)	0.572 (0.382, 0.858)	0.531 (0.328, 0.860)	0.002	
No	Ref	0.944 (0.412, 2.163)	0.427 (0.160, 1.136)	0.188 (0.073, 0.486)	<0.001	

Data are presented as OR (95% CI). Adjusted for age, gender, race, education, WBC, Neu, Hb, Plt, CKD, CVD, smoking status, hypertension, and dyslipidemia.

WBC, white blood cells; Neu, neutrophil; Hb, hemoglobin; Plt, platelet; CKD, chronic kidney disease; CVD, cardiovascular disease; OBS, oxidative balance score.

### 3.4 RCS analysis

**Figure 2:**
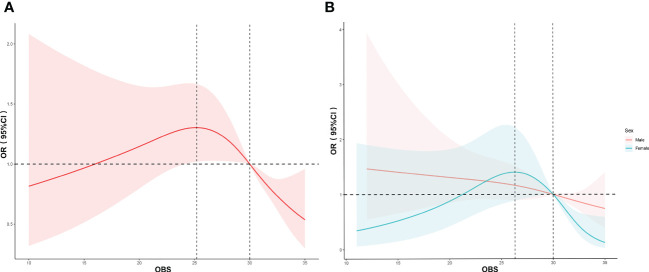
RCS analysis of the association between OBS and diabetes. The association was adjusted for age, gender, race, education, WBC count, Neu count, Hb level, Plt count, CKD, CVD, smoking status, hypertension, and dyslipidemia. The median OBS was chosen as the reference. **(A)** RCS curve of the association between OBS and diabetes among all participants. **(B)** RCS curve of the association between OBS and diabetes among female and male participants. RCS, restricted cubic spline; WBC, white blood cells; Plt, platelet; Neu, neutrophil; Lym, lymphocyte; Hb, hemoglobin; OR, odds ratio; CI, confidence interval.

The associations between OBS and diabetes in men and women were further evaluated using the RCS curves and the multivariable logistic regression (model 3). First, we found an inverted-U relationship (*p* for non-linear = 0.0118) between OBS and the risk of diabetes (**Figure 2A**). The turning point appeared around the OBS of 25.20, and the median number was 30.00. The risk of diabetes slightly increased at the beginning and then declined with the OBS after reaching the turning point. Results of the RCS analysis by gender revealed that OBS was negatively correlated with the incidence of diabetes in male individuals, and it showed a linear relationship (*p* for non-linear = 0.7816). Consistent with the relationship between the overall OBS and diabetes, we found a non-linear inverted-U relationship (*p* for non-linear < 0.001) between OBS and diabetes in female individuals (**Figure 2B**). The turning point appeared around the OBS of 26.32, and the median number was 30.00. After the OBS of 30 was reached, the risk of diabetes decreased with the increase of OBS, and the decrease in diabetes risk was more pronounced in women than in men. It was also consistent with the results of the subgroup analysis.

## 4 Discussion

For the first time, our large-scale cross-sectional study evaluated the association of OBS with diabetes based on NHANES (from 2007 to March 2020). In this study, we confirmed that the OBS and lifestyle OBS in participants without diabetes were significantly higher than those in participants with diabetes. Higher OBS and lifestyle OBS were associated with decreased risk of diabetes. After confounding factors were adjusted, it was shown that the effect of OBS on diabetes significantly relied on gender. In women and all participants, the association between OBS and diabetes showed an inverted-U relationship. In men, there was a linear relationship between OBS and the risk of diabetes.

Numerous clinical and animal studies linked oxidative stress to diabetes incidence and progression. Oxidative stress occurs when the amount of reactive oxygen species (ROS) exceeds the neutralizing capacity of antioxidants (10). Oxidative stress can interfere with the oxidation–reduction reactions in glycolysis and the electron transport chain, causing hyperglycemia. Oxidative stress activates the secondary pathways of glucose metabolism, such as glucose autoxidation and the polyol pathway, which leads to excessive ROS production and lipid peroxidation, triggering oxidative stress and exacerbating hyperglycemia (5, 10–12). Previous studies have not evaluated the relationship between OBS and diabetes, and most of them focused on the relationship between a single component of OBS and diabetes risk. Oxidative stress is a major contributor to the progression of diabetes, but not all strategies for reducing ROS are protective against diabetes (13–15). Several reasons may account for this difference. First, it is not easy to determine the effects of individual oxidative stress-related components on blood glucose control. Second, pro-oxidants or antioxidants may have antagonistic and synergistic interactions. Special attention should also be paid to the properties of the antioxidants themselves. Antioxidants can exert a pro-oxidant effect in high doses, have poor solubility and low permeability in biomembranes, and lack stability and specificity of action (16). Hence, we employed OBS as a comprehensive evaluation metric to measure the oxidative balance in an individual and investigate its influence on diabetes. Our study demonstrated that OBS was significantly higher in the non-diabetic group than in the diabetic group, and higher OBS predicted a lower risk of diabetes. RCS analysis showed that the relationship between OBS and diabetes mellitus was an inverted-U relationship. Our findings are consistent with previous epidemiological evidence indicating a significant association between oxidative stress and diabetes (5, 10, 17).

Similar to previous studies, subgroup analysis and RCS analysis revealed that gender significantly affected the correlations between OBS and diabetes. Studies have shown that the serum glucose level and oxidative stress of female diabetic rats were lower than those of male diabetic rats. At the same time, female diabetic rats had lower hydrogen peroxide levels and xanthine oxidase activity (18). Estrogen has antioxidant properties and can enhance the activity of antioxidants (19, 20). On the contrary, androgen can promote ROS production and induce oxidative stress (21). Studies have shown that estrogen, as a protective factor, can reduce the risk of insulin resistance and diabetes in women, while androgen has the opposite role (22, 23). It is worth noting that the risk of diabetes decreased more rapidly in women than in men when OBS was greater than 30. It can be concluded that it is more efficient in combating oxidative stress to keep OBS within a certain range, resulting in better glycemic control in patients with diabetes. Our results may provide new policies to reduce the burden of diabetes complications and improve the management of diabetes. Several factors such as diet, lifestyle, and genetic factors can lower the risk of chronic diseases by regulating oxidation and reducing ROS generation (24). Recently, researchers suggested that the risk of chronic disease can be reduced through lifestyle interventions (25). The risk of diabetes can be significantly reduced by lifestyle modifications, especially in male subjects (26). The mechanisms underlying the gender-specific associations merit further investigation (27). As reported in diabetic patients, female patients have lower overall muscle mass and physical function, poorer health conditions, and higher prevalence of depression, which indicate that gender-specific differences in diabetes deserve more attention (28). Thus, the lower overall muscle mass in women with diabetes may be related to OBS, especially lifestyle OBS. A study found that decreased estrogen levels in postmenopausal women increased insulin resistance and elevated the risk of diabetes (29).

The study has several advantages. First, our study found the association between OBS and diabetes for the first time and uncovered the gender-specific effects of OBS on the prevalence of diabetes. Second, the NHANES used a stratified, multistage sampling method, which increases the generalizability of our findings to non-institutionalized populations. Third, this study adjusted the results for several confounders. In addition, there are several limitations to this study. Even though we controlled for potential confounders, the role of unknown or unmeasured confounders cannot be ruled out. However, the cross-sectional nature of our study makes it difficult to infer causality. To increase the utility of our findings, the predictive value of OBS in diabetes needs to be further verified through prospective studies. Finally, dietary OBS was not significantly different between diabetic and non-diabetic groups in our study; therefore, the effects of dietary OBS in predicting diabetes risk remain unclear.

## 5 Conclusion

In conclusion, this cross-sectional study indicated that OBS, especially lifestyle OBS, was negatively associated with the prevalence of diabetes. OBS had an inverted-U relationship with the prevalence of diabetes in nationally representative adults of the USA. In addition, we found that the negative correlation between OBS and diabetes was clearer among female participants than in male participants.

## Data availability statement

Publicly available datasets were analyzed in this study. This data can be found here: https://www.cdc.gov/nchs/nhanes/index.htm.

## Ethics statement

The studies involving human participants were reviewed and approved by National Center for Health Statistics Ethics Review Board Approval. The patients/participants provided their written informed consent to participate in this study.

## Author contributions

CW and CR conceptualized this study; performed the literature search, study design, data curation, data analysis, and data interpretation; and drafted the original manuscript. YS participated in the study design and critically revised the manuscript. YS, HG, XP and LZ conceived the study and participated in study design, coordination, data collection, and analysis. All authors contributed to the article and approved the submitted version.
