# Supplementary material for: Gender-specific effects of oxidative balance score on the prevalence of diabetes in the US population from NHANES
Source: Front Endocrinol (Lausanne). 2023 May 4;14:1148417. doi: 10.3389/fendo.2023.1148417 (PMC10194026; doi:10.3389/fendo.2023.1148417)
Supplement: Supplementary file 1 [file Table_1.docx]

Supplementary Material

**Gender-specific effects of Oxidative Balance Score on the Prevalence of Diabetes in the** **US Population from NHANES**

**Cui-ling Wu*^1†^, Chen-xia Ren^2†^, Ying-da Song^3,4^, Hui-fang Gao^1^, Xin Pang^1^, Lian-yun Zhang^1^**

^†^These authors contributed equally to this work

*** Correspondence:** Cuiling Wu: [clw928@czmc.edu.cn](mailto:clw928@czmc.edu.cn)

# Supplementary Data

Supplementary Material include supplementary table 1-supplementary table 4. The above documents were submitted separately.

# Supplementary Tables

**Supplemental table 1.** Baseline characteristics of all participants by the dietary oxidative balance score (dietary OBS) quartile.

| **Variables** | **Overall** | **Q1** | **Q2** | **Q3** | **Q4** | ***P* value** |
| --- | --- | --- | --- | --- | --- | --- |
| Wbc (×10^9^/L) | 7.01(0.05) | 7.32(0.10) | 7.16(0.08) | 6.90(0.09) | 6.68(0.07) | < 0.0001 |
| Neu (×10^9^/L) | 4.12(0.04) | 4.33(0.08) | 4.24(0.06) | 4.02(0.07) | 3.88(0.05) | < 0.0001 |
| Lym (×10^9^/L) | 2.10(0.01) | 2.17(0.03) | 2.12(0.03) | 2.09(0.02) | 2.04(0.03) | 0.03 |
| Hb (g/L) | 14.52(0.03) | 14.60(0.06) | 14.53(0.04) | 14.47(0.05) | 14.47(0.06) | 0.31 |
| Plt (×10^6^/L) | 237.68(1.57) | 243.60(2.23) | 238.39(2.31) | 239.08(2.68) | 230.11(2.88) | 0.003 |
| OBS | 29.69(0.08) | 24.88(0.17) | 29.58(0.06) | 31.27(0.06) | 32.60(0.06) | < 0.0001 |
| Age(years) | 45.23(0.41) | 43.36(0.65) | 44.27(0.61) | 45.06(0.63) | 48.22(0.69) | < 0.0001 |
| lifestyle OBS | 4.13(0.04) | 3.70(0.07) | 4.00(0.06) | 4.27(0.06) | 4.54(0.06) | < 0.0001 |
| DM, n (%) |  |  |  |  |  | 0.38 |
| no | 4611(88.11) | 1147(89.58) | 1360(90.29) | 1024(91.66) | 1080(91.98) |  |
| yes | 622(11.89) | 194(10.42) | 173( 9.71) | 122( 8.34) | 133( 8.02) |  |
| CKD, n (%) |  |  |  |  |  | 0.1 |
| no | 4693(89.68) | 1174(89.04) | 1388(91.21) | 1034(92.62) | 1097(92.05) |  |
| yes | 540(10.32) | 167(10.96) | 145( 8.79) | 112( 7.38) | 116( 7.95) |  |
| CVD, n (%) |  |  |  |  |  | 0.94 |
| no | 4910(93.83) | 1235(94.81) | 1445(94.93) | 1090(94.91) | 1140(94.34) |  |
| yes | 323(6.17) | 106(5.19) | 88(5.07) | 56(5.09) | 73(5.66) |  |
| Hypertension, n (%) |  |  |  |  |  | 0.2 |
| no | 3485(66.6) | 823(65.62) | 1053(71.32) | 791(70.43) | 818(68.72) |  |
| yes | 1748(33.4) | 518(34.38) | 480(28.68) | 355(29.57) | 395(31.28) |  |
| Hyperlipidemia, n (%) |  |  |  |  |  | 0.56 |
| no | 1841(35.18) | 466(33.96) | 536(32.49) | 429(35.45) | 410(32.55) |  |
| yes | 3392(64.82) | 875(66.04) | 997(67.51) | 717(64.55) | 803(67.45) |  |
| Smoking status, n (%) |  |  |  |  |  | < 0.0001 |
| never | 2570(49.11) | 550(41.19) | 753(49.62) | 621(54.68) | 646(54.48) |  |
| former | 1364(26.07) | 274(22.19) | 403(27.99) | 302(28.29) | 385(33.15) |  |
| now | 1299(24.82) | 517(36.62) | 377(22.39) | 223(17.03) | 182(12.37) |  |
| Age group, n (%) |  |  |  |  |  | < 0.0001 |
| 20-39 | 2103(40.19) | 552(43.61) | 658(42.01) | 469(39.69) | 424(31.57) |  |
| 40-59 | 1951(37.28) | 499(41.84) | 565(40.17) | 439(42.49) | 448(42.44) |  |
| 60-79 | 1179(22.53) | 290(14.55) | 310(17.82) | 238(17.82) | 341(25.98) |  |
| PIR |  |  |  |  |  | < 0.0001 |
| <=1 | 812(15.52) | 289(13.69) | 245( 9.84) | 148( 7.62) | 130( 6.67) |  |
| >1 | 4421(84.48) | 1052(86.31) | 1288(90.16) | 998(92.38) | 1083(93.33) |  |
| Education, n (%) |  |  |  |  |  | < 0.0001 |
| college and higher | 3495(66.79) | 712(59.66) | 1000(70.90) | 838(79.09) | 945(84.09) |  |
| middle and high school | 1535(29.33) | 559(37.81) | 466(27.14) | 276(19.82) | 234(15.02) |  |
| primary school and less | 203(3.88) | 70(2.54) | 67(1.97) | 32(1.09) | 34(0.89) |  |
| Race, n (%) |  |  |  |  |  | < 0.0001 |
| black | 1029(19.66) | 414(15.07) | 282( 7.67) | 159( 5.31) | 174( 5.13) |  |
| mexican | 623(11.91) | 150(6.11) | 199(6.49) | 161(6.85) | 113(4.30) |  |
| other | 989(18.9) | 221(10.71) | 268( 9.60) | 217(10.34) | 283(12.69) |  |
| white | 2592(49.53) | 556(68.10) | 784(76.23) | 609(77.49) | 643(77.88) |  |
| Gender, n (%) |  |  |  |  |  | 0.34 |
| Female | 2062(39.4) | 481(38.23) | 612(42.71) | 480(42.88) | 489(42.53) |  |
| Male | 3171(60.6) | 860(61.77) | 921(57.29) | 666(57.12) | 724(57.47) |  |

All values represented are weighted means (standard deviation), or counts (weighted percentage). Abbreviations: SD, standard deviation; PIR, poverty index ratio; Wbc, white blood cells; Neu, neutrophil; Lym, lymphocyte; Hb, hemoglobin; Plt, platelet; CKD,chronic kidney disease; CVD, cardiovascular disease; OBS, oxidative balance score; DM, diabetes.

**Supplemental table 2.** Baseline characteristics of all participants by the lifestyle oxidative balance score (lifestyle OBS) quartile.

| **Variables** | **Overall** | **Q1** | **Q2** | **Q3** | **Q4** | **Pvalue** |
| --- | --- | --- | --- | --- | --- | --- |
| Wbc (×10^9^/L) | 7.01(0.05) | 7.57(0.08) | 7.08(0.07) | 6.63(0.07) | 6.43(0.09) | < 0.0001 |
| Neu (×10^9^/L) | 4.12(0.04) | 4.47(0.06) | 4.16(0.05) | 3.88(0.06) | 3.75(0.08) | < 0.0001 |
| Lym (×10^9^/L) | 2.10(0.01) | 2.25(0.03) | 2.12(0.02) | 2.00(0.02) | 1.94(0.03) | < 0.0001 |
| Hb (g/L) | 14.52(0.03) | 14.69(0.05) | 14.58(0.06) | 14.34(0.05) | 14.36(0.05) | < 0.0001 |
| Plt (×10^6^/L) | 237.68(1.57) | 241.47(2.05) | 237.57(2.24) | 237.02(3.21) | 232.25(2.75) | 0.05 |
| OBS | 29.69(0.08) | 27.26(0.12) | 29.57(0.10) | 30.84(0.13) | 32.59(0.10) | < 0.0001 |
| dietary OBS | 25.55(0.06) | 24.86(0.12) | 25.57(0.10) | 25.84(0.13) | 26.38(0.10) | < 0.0001 |
| Age(years) | 45.23(0.41) | 44.17(0.57) | 45.07(0.58) | 46.52(0.66) | 45.71(0.87) | 0.03 |
| DM, n (%) |  |  |  |  |  | < 0.0001 |
| no | 4611(88.11) | 1522(86.65) | 1271(92.62) | 998(91.34) | 820(94.91) |  |
| yes | 622(11.89) | 293(13.35) | 146( 7.38) | 119( 8.66) | 64( 5.09) |  |
| CKD, n (%) |  |  |  |  |  | 0.07 |
| no | 4693(89.68) | 1602(89.21) | 1274(92.66) | 1014(92.44) | 803(91.37) |  |
| yes | 540(10.32) | 213(10.79) | 143( 7.34) | 103( 7.56) | 81( 8.63) |  |
| CVD, n (%) |  |  |  |  |  | 0.4 |
| no | 4910(93.83) | 1686(93.91) | 1324(94.79) | 1059(94.90) | 841(95.94) |  |
| yes | 323(6.17) | 129(6.09) | 93(5.21) | 58(5.10) | 43(4.06) |  |
| Hypertension, n (%) |  |  |  |  |  | < 0.0001 |
| no | 3485(66.6) | 1064(61.77) | 942(67.08) | 796(72.38) | 683(80.98) |  |
| yes | 1748(33.4) | 751(38.23) | 475(32.92) | 321(27.62) | 201(19.02) |  |
| Hyperlipidemia, n (%) |  |  |  |  |  | < 0.0001 |
| no | 1841(35.18) | 532(25.95) | 516(34.53) | 412(32.92) | 381(45.58) |  |
| yes | 3392(64.82) | 1283(74.05) | 901(65.47) | 705(67.08) | 503(54.42) |  |
| Smoking status, n (%) |  |  |  |  |  | < 0.0001 |
| never | 2570(49.11) | 594(32.06) | 627(45.41) | 719(64.37) | 630(70.71) |  |
| former | 1364(26.07) | 435(26.43) | 387(30.08) | 295(27.60) | 247(28.33) |  |
| now | 1299(24.82) | 786(41.51) | 403(24.51) | 103( 8.03) | 7( 0.96) |  |
| Age group, n (%) |  |  |  |  |  | 0.04 |
| 20-39 | 2103(40.19) | 753(40.61) | 563(38.27) | 435(37.08) | 352(40.90) |  |
| 40-59 | 1951(37.28) | 715(44.02) | 543(42.75) | 398(40.87) | 295(37.03) |  |
| 60-79 | 1179(22.53) | 347(15.37) | 311(18.98) | 284(22.04) | 237(22.07) |  |
| PIR |  |  |  |  |  | < 0.0001 |
| <=1 | 812(15.52) | 383(13.35) | 228( 9.44) | 129( 7.00) | 72( 5.43) |  |
| >1 | 4421(84.48) | 1432(86.65) | 1189(90.56) | 988(93.00) | 812(94.57) |  |
| Education, n (%) |  |  |  |  |  | < 0.0001 |
| college and higher | 3495(66.79) | 1046(62.15) | 896(71.19) | 816(79.40) | 737(89.53) |  |
| middle and high school | 1535(29.33) | 705(36.12) | 453(26.58) | 257(19.41) | 120( 9.41) |  |
| primary school and less | 203(3.88) | 64(1.73) | 68(2.23) | 44(1.19) | 27(1.06) |  |
| Race, n (%) |  |  |  |  |  | < 0.0001 |
| black | 1029(19.66) | 529(13.16) | 257( 7.59) | 172( 6.15) | 71( 2.76) |  |
| mexican | 623(11.91) | 206(6.32) | 191(6.77) | 145(6.84) | 81(3.14) |  |
| other | 989(18.9) | 265(10.18) | 260( 9.90) | 219(10.28) | 245(13.66) |  |
| white | 2592(49.53) | 815(70.34) | 709(75.74) | 581(76.73) | 487(80.44) |  |
| Gender, n (%) |  |  |  |  |  | 0.11 |
| Female | 2062(39.4) | 698(39.76) | 541(39.98) | 450(42.55) | 373(46.49) |  |
| Male | 3171(60.6) | 1117(60.24) | 876(60.02) | 667(57.45) | 511(53.51) |  |

All values represented are weighted means, or counts.

**Supplemental table 3.** Association of the dietary oxidative balance score (dietary OBS) with diabetes, NHANES 2007-march 2020.

| **Diabetes** | **OR(95%CI);*p*-Value** | | | |
| --- | --- | --- | --- | --- |
|  | Crude model | Model 1 | Model 2 | Model 3 |
| Continuous | 0.81(0.74,0.88) <0.0001 | 0.77(0.70,0.84) <0.0001 | 0.78(0.72,0.86) <0.0001 | 0.82(0.74,0.91) <0.001 |
| Q1 | 1.00 (ref). | 1.00 (ref). | 1.00 (ref). | 1.00 (ref). |
| Q2 | 0.52(0.37,0.72) <0.001 | 0.46(0.33,0.65) <0.0001 | 0.47(0.34,0.66) <0.0001 | 0.49(0.35,0.69) <0.0001 |
| Q3 | 0.62(0.44,0.86) 0.005 | 0.54(0.37,0.77) <0.001 | 0.57(0.39,0.82) 0.003 | 0.64(0.43,0.96) 0.03 |
| Q4 | 0.35(0.21,0.58) <0.0001 | 0.30(0.18,0.51) <0.0001 | 0.32(0.19,0.54) <0.0001 | 0.39(0.22,0.67) <0.001 |
| *P* for trend | <0.0001 | <0.0001 | <0.0001 | <0.001 |

The dietary oxidative balance score was converted from a continuous variable to a categorical variable (Quartiles). Data are presented as OR (95% CI). Crude model was adjusted no covariates. Model 1 was adjusted for age, sex, race, and education; Model 2 was adjusted for Model 1 + Wbc, Neu, Hb, and Plt. Model 3 was adjusted for Model 2 + CKD, CVD, smoking status, hypertension, and hyperlipidemia. Abbreviations: NHANES: the National Health and Nutrition Examination Survey; Wbc, white blood cells; Plt, platelet; Neu, neutrophil; Lym, lymphocyte; Hb, hemoglobin; OR, odds ratio; CI, confidence interval.

**Supplemental table 4.** Association of the lifestyle oxidative balance score (lifestyle OBS) with diabetes, NHANES 2007-march 2020.

| **Diabetes** | **OR(95%CI),*p*-Value** | | | |
| --- | --- | --- | --- | --- |
|  | Crude model | Model 1 | Model 2 | Model 3 |
| Continuous | 0.97(0.94,1.00) 0.04 | 0.97(0.94,1.00) 0.05 | 0.98(0.95,1.01) 0.16 | 0.99(0.96,1.02) 0.45 |
| Q1 | 1.00(reference) | 1.00(reference) | 1.00(reference) | 1.00(reference) |
| Q2 | 0.92(0.67,1.28) 0.63 | 0.97(0.69,1.36) 0.85 | 0.97(0.69,1.36) 0.84 | 1.05(0.73,1.52) 0.78 |
| Q3 | 0.78(0.54,1.13) 0.19 | 0.82(0.55,1.22) 0.32 | 0.86(0.57,1.29) 0.45 | 0.97(0.63,1.49) 0.88 |
| Q4 | 0.75(0.51,1.11) 0.15 | 0.67(0.47,0.97) 0.03 | 0.70(0.48,1.02) 0.06 | 0.80(0.55,1.15) 0.22 |
| *P* for trend | 0.08 | 0.02 | 0.04 | 0.17 |

The lifestyle oxidative balance score was converted from a continuous variable to a categorical variable (Quartiles). Data are presented as OR (95% CI). Crude model was adjusted no covariates. Model 1 was adjusted for age, sex, race, and education; Model 2 was adjusted for Model 1 + Wbc, Neu, Hb, and Plt. Model 3 was adjusted for Model 2 + CKD, CVD, smoking status, hypertension, and hyperlipidemia. Abbreviations: NHANES: the National Health and Nutrition Examination Survey; Wbc, white blood cells; Plt, platelet; Neu, neutrophil; Lym, lymphocyte; Hb, hemoglobin; OR, odds ratio; CI, confidence interval.
